# Incidence, prognosis, and perinatal outcomes of and risk factors for severe twin–twin transfusion syndrome with right ventricular outflow tract obstruction in the recipient twin after fetoscopic laser photocoagulation

**DOI:** 10.1186/s12884-022-04668-1

**Published:** 2022-04-15

**Authors:** Yao-Lung Chang, An-Shine Chao, Shuenn-Dyh Chang, Po-Jen Cheng, Wen-Fang Li, Chin-Chieh Hsu

**Affiliations:** Department of Obstetrics and Gynecology, Chang Gung Memorial Hospital, Chang Gung University College of Medicine, 5, Fu-Shin Street, Kweishan, Taoyuan, Taiwan, ROC 333

**Keywords:** Twin-to-twin transfusion syndrome, Pulmonary stenosis, Pulmonary atresia, Recipient twin, Fetal laser photocoagulation

## Abstract

**Background:**

Right ventricular outflow tract obstruction (RVOTO) is the most frequently encountered congenital heart disease in patients with twin –twin transfusion syndrome (TTTS) and is especially prevalent in the recipient twin. In this retrospective study, we evaluated the incidence, prognosis, postnatal management, and perinatal outcomes of and risk factors for RVOTO in the recipient twin in severe TTTS cases which diagnosed before 26 weeks after fetoscopic laser photocoagulation (FLP) at a single center in Taiwan.

**Methods:**

RVOTO was diagnosed using fetal or postnatal echocardiography. The fetal outcomes evaluated were perinatal survival rate, neonatal brain image anomalies rate, gestational age at delivery, and birth weight.

**Results:**

Total 187 severe TTTS cases were included; 14 (7.49%) had a recipient twin with RVOTO (12 cases of pulmonary stenosis and 2 of pulmonary atresia). Of these 14 cases, 3 (21.4%) demonstrated improvements in outflow obstruction after FLP, and 11 (78.6%) resulted in perinatal survival. Of the 11 survivors, 5 (45.5%) received transcatheter balloon valvuloplasty to alleviate the RVOTO. The perinatal survival rate, gestational age at delivery, neonatal brain image anomaly rate, and birth weights did not significantly differ between the groups in which the recipient twin had versus did not have RVOTO. Generally, the recipient twin had RVOTO received FLP at a younger gestational age (in weeks; 19.3 ± 2.4 vs. 20.7 ± 2.6, *p* = 0.048) and had a higher percentage of cases at Quintero stage IV (50.0% vs. 12.1%, *p* < 0.001) than those in which the recipient twin did not have with RVOTO. Using logistic regression, we discovered that FLP at a younger gestational age (*p* = 0.046, odds ratio = 0.779) and TTTS at Quintero stage IV (*p* = 0.001, odds ratio = 7.206) were risk factors for the recipient twin developing RVOTO after FLP in severe TTTS cases.

**Conclusions:**

The post-FLP perinatal outcomes of cases of severe TTTS in which the recipient twin had versus did not have RVOTO were comparable in this study, which may have been due to the similar gestational ages at delivery and strong influence of high Quintero stages (stages III and IV).

## Background

Twin–twin transfusion syndrome (TTTS) is a complication that occurs in approximately 9% of monochorionic diamniotic twin pregnancies [1] due to unbalanced intertwin placenta flow with preferential shunting of blood from one twin (donor) to the other (recipient) through vascular communication. As discussed by Quintero et al. [2], TTTS is clinically characterized by concurrent polyhydramnios in the “recipient” twin and oligohydramnios in the “donor” twin, as identified through ultrasound. Generally, the donor twin exhibits hypovolemia, and the recipient exhibits hypervolemia. To compensate for the hypovolemia, the donor twin secretes vasoactive mediators, such as endothelin I and renin, which circulate to the recipient twin through intertwin anastomoses; the vasoactive substances can cause polyhydramnios, cardiomyopathy, and atrioventricular valve regurgitation in the recipient twin [3–5]. In 70% of TTTS cases, the recipient twin demonstrates echocardiographic signs of cardiovascular complications at the time of diagnosis [6, 7].

In TTTS survivors, the incidence of congenital heart disease can be as high as 87 per 1000 live births, a 12-fold increased incidence compared with that for singletons [8]. However, the mechanism underlying congenital heart disease development in cases of TTTS is not completely understood; multiple factors have been proposed, such as hemodynamic derangements in the donor and recipient twins, genetic predisposition in twinning, and abnormal placentation [9]. Right ventricular outlet obstruction (RVOTO), such as in pulmonary stenosis (PS) and pulmonary atresia (PA), is the most prevalent form of congenital heart disease, with a reported incidence of 6.7%–12.9% in recipient twins [10–13]. This higher risk exists because recipient twins have high risks of right heart dysfunction, cardiomegaly, and tricuspid valve regurgitation, which can cause the flow through the pulmonary valve to decrease, potentially leading to narrowing and stenosis of the fetal pulmonary valve of the recipient twin [6]. The etiology of the higher incidence of cardiomyopathy and the lower pulmonary valve flow in the recipient twin compared with the donor twin has been hypothesized as being the result of differing levels of renin–angiotensin activity between the recipient and donor twins [14]. The recipient twin may have higher renin–angiotensin activity than the donor twin, leading higher incidence of RVOTO in recipient twins [15]. Differences in volume overload, with volume overload occurring more frequently in recipient than in donor twins due to intermittent absent or reversed umbilical artery end-diastolic flow, have also been reported to increase the incidence of recipient-twin RVOTO [16]. However, the true etiology of RVOTO in recipient twins remains unclear [17].

Fetoscopic laser photocoagulation (FLP) is the first-line therapy for TTTS diagnosed prior to 26 weeks of gestation [18–20]. Because FLP can interrupt intertwin anastomoses, it blocks the vasoactive mediators from the donor to the recipient twin. Although regression has been achieved following FLP in many cases of RVOTO in recipient twins [17, 21, 22], the incidence of RVOTO in recipient twins after FLP remains high [8]. Furthermore, persistent RVOTO in recipient twins with TTTS after FLP has been reported to be associated with decreased survival of fetuses [11, 17] and a less favorable neonatal outcome in cases of PA [10]. Furthermore, a study demonstrated that RVOTO in recipient twins has no effect on donor twin survival [13].

Our institution is the largest laser center for treatment of TTTS cases in Taiwan. In this study, we included cases of severe TTTS ( those diagnosed before 26 weeks of gestation) to analyze the incidence, prognosis, and postnatal management of and the risk factors for RVOTO in recipient twins and the influence of RVOTO in recipient twins on perinatal outcomes, including survival rate, gestational age of delivery, birth weight, and neonatal brain image results.

## Methods

This retrospective study was performed at the Chang Gung Memorial Hospital of Taoyuan, Taiwan, and it was approved by the Institutional Review Board of the Chang Gung Medical Foundation (202101274B0). The diagnosis of TTTS was based on ultrasound findings, as defined by Quintero et al. [2]. We included TTTS cases from October 2007 to March 2021 diagnosed before the gestational age of 26 weeks and that were treated with FLP at our hospital. The FLP procedure for TTTS has been described previously [23].

A complete evaluation of the fetal structure was conducted with a Voluson Expert 8 (GE Medical Systems, Milwaukee, WI, USA) to assess the fetal cardiac anatomy in all patients with TTTS before FLP. Color and pulsed-wave Doppler measurements were obtained in the absence of fetal breathing and movements. All cases of suspected PS or PA at the diagnosis of TTTS were referred for fetal echocardiography performed by a perinatal cardiologist. The diagnosis of PS was based on high peak systolic velocity (>90 cm/s) and turbulent flow across the pulmonary valve, with or without thickened and domed pulmonary valve cusps, which are associated with structural or functional right heart abnormalities. PA was diagnosed in the absence of forward flow across the pulmonary valve during systolic and reverse flow in the ductus arteriosus [24]. Cases associated with major cardiac anomalies other than PS and PA, such as Ebstein’s anomaly and tetralogy of fallot (TOF), or genetic anomalies, such as trisomy, were excluded from this study. Patients with TTTS were followed up with serial ultrasound 1 day before and every day after laser therapy if the patient was admitted. In cases of ultrasound-based PS or PA diagnosis, the condition of the fetal cardiac structure of the recipient twins was followed up in the outpatient department at every visit.

After a delivery in which the recipient twin survived, the pulmonary valve pathology was confirmed in the recipient twin through postnatal echocardiography or angiographic examinations if transcatheter balloon valvuloplasty (TBV) was performed. Perinatal survival was defined as survival 28 days after delivery.

Because fetal survival and intact neurological status are key outcomes for fetuses with TTTS after FLP, all living neonates with TTTS who had undergone FLP received cranial ultrasound examinations within 1 month of delivery. If two or more cranial ultrasound tests were performed, the most recent result was used. Mild cerebral image anomalies were defined as the presence of at least one of the following: intraventricular hemorrhage (IVH) of grade I or II; lenticulostriate vasculopathy; and subependymal pseudocysts. Severe cerebral image anomalies were defined as the presence of at least one of the following: IVH of grade III or IV; cystic periventricular leukomalacia of grade II or higher; porencephalic cysts; and ventricular dilatation. Ventricular dilatation was considered present when the width of the unilateral or both lateral ventricles exceeded that in the 97th percentile [23].

Statistical analysis was conducted using SPSS (version 11.0 for Windows; SPSS, Chicago, IL, USA). The normality of the data was assessed using the Shapiro–Wilk test. The Student’s *t* test or Mann–Whitney U test was used to compare continuous variables between groups. Qualitative data were compared using the χ^2^ test or Fisher’s exact test. A *p* value of less than 0.05 was considered significant. Logistic regression (forward condition) was used to determine the RVOTO risk factors in recipient twins. The gestational age at FLP, Quintero stage, maternal age at operation, and maximum vertical pocket of amniotic fluid (MVP) were included in to the mode when the *p* value for the variable was less than 0.05, and the variables were removed when the *p* value was greater than 0.1. The odds ratios of the variables are expressed as the odds (95% confidence interval).

## Results

**Fig. 1:**
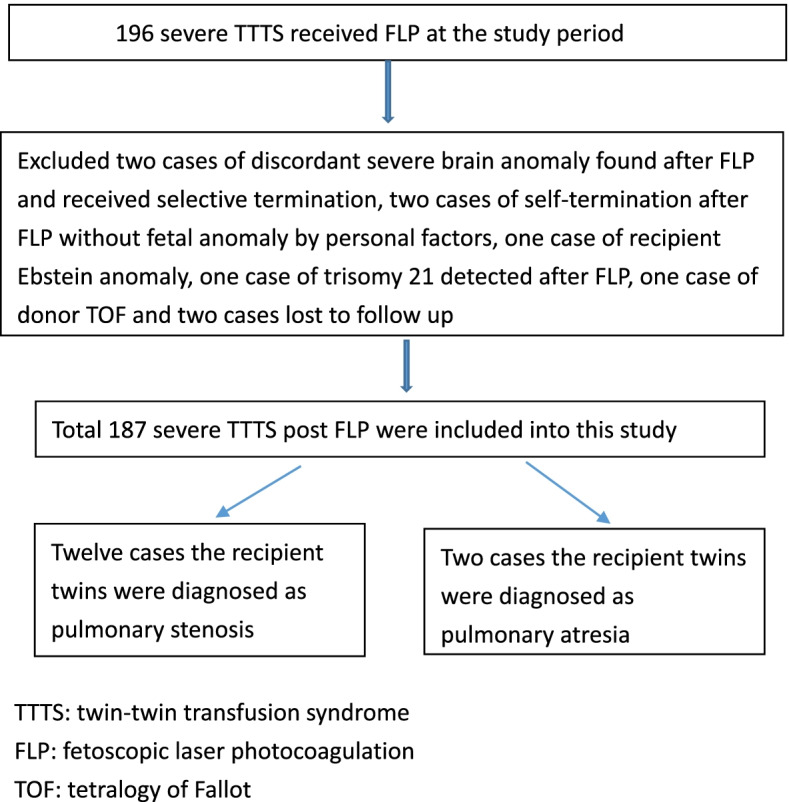
The flowchart of severe TTTS received FLP included and excluded into this study. TTTS: twin-twin transfusion syndrome. FLP: fetoscopic laser photocoagulation. TOF: tetralogy of Fallot

After exclusion of two cases with selective termination after laser therapy due to discordant fetal major brain image anomaly in one fetus, two cases of self-termination due to personal factors, one case of Ebstein’s anomaly in the recipient twin, one case of trisomy 21 detected after FLP, one case of donor TOF, and two cases lost to follow-up, a total of 187 cases of severe TTTS receiving FLP were included (Figure 1). The preoperational characteristics and fetal survival rates after FLP are listed in Table 1. RVOTO was identified in the recipient twin in 14 (7.49%) of the 187 TTTS cases. Maternal age at operation, Quintero stage, interval between operation and delivery, and fetal survival did not significantly differ between the cases of TTTS with versus without recipient twins with RVOTO. The gestational age upon FLP was lower among the TTTS cases in which the recipient twin had RVOTO. Although the Quintero stage distributions of the two groups were significantly different, the percentage of cases with a high Quintero stage (stage III or IV) did not significantly differ. Perinatal survival was achieved in 11 (78.6%) of the 14 cases with recipient twins with RVOTO and in 130 (75.1%) of the 173 cases without recipient twins with RVOTO, indicating a comparable rate of perinatal survival between the two groups. Three (21.4%) of the 14 cases with recipient twins with RVOTO demonstrated improvements after FLP, and in five cases (45.5%), TBV was required to alleviate the RVOTO.

**Table 1:** Characteristics of the patients with twin-to-twin transfusion syndrome patients with and without recipient right ventricular obstruction (RVOTO) before fetoscopic laser therapy

		Recipient twin with RVOTO (*N*=14)	Recipient twin without RVOTO (*n*=173)	*p*
Maternal age at operation (year-old)		33.7 ± 4.6	31.7 ± 4.5	0.31^#^
Gestational age at operation (weeks)		19.3 ± 2.4	20.7 ± 2.6	0.048^&^
Gestational age at delivery (weeks)		33.2± 5.8	31.3 ± 5.5	0.16^#^
RVOTO Regression after laser		4		
Interval from operation to delivery (days)		92.1± 48.7	74.1±41.7	0.09^#^
Quintero stage (number)				<0.001^*^
	I	1	30	
	II	5	58	
	III	1	65	
	IV	7	21	
High Quintero stage (III or IV)		57.1% ( 8/14)	49.7% (86/173)	0.78*
Donor survival (number)		12	115	0.23*
Recipient survival (number)		11	130	1.0*

#: Mann-Whitney Test

&: student t test

*: chi-square or fisher’s exact test

The birth weights of the recipient and donor twins and the results of the fetal brain imaging in the cases of severe TTTS after FLP with recipient twin perinatal survival are listed in Table 2. No significant difference was identified in the birth weights of the recipient and donor twins or in the neonatal image anomaly rates of the TTTS groups with and without recipient twins with RVOTO. The neonatal brain image anomaly rate was 3.71% (10/268) in the 141 TTTS cases with recipient twin survival (268 total surviving fetuses) after FLP, and the severe brain image anomaly rate in the 141 TTTS cases was 0.74% (2/268; Table 2).

**Table 2:** Characteristics of the patients with severe twin-to-twin transfusion syndrome with and without recipient right ventricular obstruction (RVOTO) post fetoscopic laser therapy with recipient survival

	Recipient twin with RVOTO (*N*=11)	Recipient twin without RVOTO (*n*=130)	*p*
Mean birth weight of donor (g)	1685 ± 591	1500 ± 696	0.39^#^
Mean birth weight of recipient (g)	2053 ± 646	1786 ± 646	0.14^#^
Mild brain image anomaly of donor	1/11 (9.1%)	3/130 (2.3%)	0.28*
Mild brain image anomaly of recipient	0/11	4/130 (3.1%)	1.0*
Severe brain image anomaly of donor	0/11	2 /130 (1.5%)	1.0*
Severe brain image anomaly of recipient	0/11	0/130	1.0*

Mild cerebral image anomaly: defined by lesions detected in cranial ultrasound scans with the presence of at least one of the following: intraventricular hemorrhage (IVH) grade I and II, lenticulostriate vasculopathy, and subependymal pseudocysts.

Severe cerebral image anomaly: defined by the presentation of one of the following signs: IVH grade III or grade IV, cystic periventricular leukomalacia grade II or more, porencephalic cysts, and ventricular dilatation. Ventricular dilatation was diagnosed when the width of the unilateral or of both lateral ventricles exceeded the 97th percentile.

#: Mann-Whitney Test; *: chi-square or fisher’s exact test

The individual diagnoses and brief histories of the 14 patients with severe TTTS with recipient twins with RVOTO are listed in Table 3. One patient did not receive a formal diagnosis of PS through fetal echocardiography before FLP (case 14); mild PS was identified after delivery through neonatal echocardiography.

**Table 3:** List of severe twin-twin transfusion syndrome with recipient twin right ventricular outflow tract obstruction

	Quintero stage	GA at FLT (weeks)	PD	RVOTO regression after FLT	GA at delivery (weeks)	Live birth of RT	BW of RT (g)	intervention after delivery	
1	IV	17.29	PA	no	17.86	no			PPROMs 4 days after FLT and abortion
2	I	20.71	PS	yes	32.71	yes	2390		No PS found after delivery
3	III	20.14	PS	no	27.29	no			Recipient IUFD after FLP
4	IV	19.86	PS	yes	35.71	yes	2690		No residual PS
5	II	19.0	PS	no	39.57	yes	2430	TBV	Mild PS after TBV
6	IV	17.43	PA	no	29.57	no			Recipient IUFD after FLP
7	IV	17.57	PS	no	36.57	yes			no residual PS
8	II	17.71	PS	no	37.29	yes	1580	TBV	Mild PS post TBV
9	IV	20.57	PS	yes	35.57	yes	2435		No PS found after delivery
10	II	17.14	PS	no	34.00	yes	1880	TBV	
11	II	21.0	PS	no	35.86	yes	2430		Mild PS found after delivery, resolution 6 months later
12	IV	19.14	PS	no	36.86	yes	2105	TBV	residual mild PS after TBV
13	IV	16.14	PS	no	36.14	yes	2740	TBV	residual mild PS after TBV
14	II	25.43	no	no	27.43	yes	766		Mild PS found after delivery, resolution 3 months later

*GA* Gestational age, *FLT* Fetal laser therapy, *PD* Prenatal diagnosis, *PA* Pulmonary atresia, *PA* Pulmonary stenosis, *RVOTO* Right ventricular outflow tract obstruction, *RT* Recipient twin, *BW* Birth weight, *IUFD* Intrauterine fetal death, *TBV* Trans-catheter balloon valvularplasty, *PPROMS* Premature preterm rupture of membranes

To identify the risk factors for recipient twins developing RVOTO, logistic regression was employed, with maternal age and gestational age at FLP, high Quintero stage, Quintero stage IV, and MVP of the recipient twin used as variables. Young gestational age at FLP and Quintero stage IV were identified as risk factors for a recipient twin developing RVOTO in cases of severe TTTS (Table 4).

**Table 4:** Predisposing factors for recipient RVOTO in severe TTTS cases

variable	odds ratio (95% CI)	*P*
Whether Quintero Stage IV	7.206 (2.226~23.328)	0.001
Gestational age at operation (week)	0.779 (0.609~0.996)	0.046

*RVOTO* Right ventricular outlet obstruction, *TTTS* Twin-twin transfusion syndrome

The odds ratios of the variables were expressed as odds ratio (95% confidence interval)

Results were calculated by Forward Stepwise (Conditional) model

Variables to enter the mode when the *p*-value was less than 0.05 and removed when the probability value was more than 0.1

## Discussion

The results of this study revealed that the prevalence of RVOTO in recipient twins affected by severe TTTS was 7.49%, and RVOTO was improved in 21.4% (3/14) of recipient twins after FLP. Furthermore, the incidence of RVOTO in recipient twins with perinatal survival after FLP was 7.8% (11/141), and 45.5% (5/11) of the surviving recipient twins with RVOTO required TBV to alleviate outflow obstruction symptoms. The post-FLP prognoses of severe TTTS in cases of a recipient twin with versus without RVOTO were comparable, and the two groups had similar neonatal survival rates, gestational ages at delivery, birth weights, and brain image anomaly rates.

The prevalence of congenital heart disease in the general population is approximately 1% [25], and the prevalence of RVOTO ranges from 0.05% to 0.15% [26]. In our study, the incidence of RVOTO in recipient twins for severe TTTS cases was 7.49% before FLP and 7.8% after delivery, much higher incidence than that in the general public. These findings indicate that fetal echocardiography should be performed for fetuses with TTTS to identify any cardiac anomalies. However, because the post-FLP outcomes of TTTS in which the recipient twin has RVOTO are comparable with those of TTTS in which the recipient twin does not have RVOTO and because RVOTO can improve after FLP, a prenatal diagnosis of RVOTO in a TTTS recipient twin is probably not a poor prognostic factor and is not an indicator that FLP should not be administered. FLP was reported to improve the pulmonary valve flow in recipient twins; this is probably because FLP improves right ventricular systolic and diastolic functions [27]. However, the prevalence of TTTS with congenital heart disease at birth may be influenced by the survival rate of patients with TTTS being improved after treatment with FLP [8, 28]. FLP may not reduce the incidence of RVOTO in surviving recipient twins because the survival rate of recipient twins both with and without RVOTO generally improves with application of FLP. Moreover, the pediatric cardiac echography–detected 7.8% (11/141) incidence of RVOTO after FLP in the surviving recipient twins of our study further emphasizes the importance of pediatric cardiac echography for survivors of TTTS.

The prevalence of prenatal RVOTO occurring only after laser therapy was reported as 5.6% [10]. As was true of our case 14 (Table 3), in such cases RVOTO can only be identified after delivery, and therefore, RVOTO may develop after laser therapy. We generally check the fetal color Doppler echocardiograms of fetuses with TTTS before and after FLP. However, in clinical follow-ups after FLP, two-dimensional echocardiography is performed only in cases without prelaser echocardiography and immediately after laser RVOTO diagnosis. Therefore, mild PS that did not develop immediately after FLP may be overlooked. Moreover, if mild PS develops after FLP and subsides before delivery, we may not discover the PS through pediatric cardiography. Consequently, the incidence of RVOTO in recipient twins after FLP may be higher than we report.

Onset of TTTS early in gestation was reported as a risk factor for RVOTO in TTTS recipient twins, which may have been due to increased vulnerability to hemodynamic imbalances in the fetal heart in early pregnancy [29]. We also discovered that recipient twins developed RVOTO more often when TTTS was diagnosed as Quintero stage IV and FLP was performed at younger gestational age. In our institution, we generally treat cases of TTTS within 1 or 2 days of diagnosis. Therefore, in our institution, young gestational age upon FLP also indicates young gestational age at diagnosis. Our data revealed the prevalence of recipient RVOTO to be highest in cases with Quintero stage IV, which supports the findings of a report also demonstrating that the rate of recipient twins developing RVOTO was higher in cases of Quintero stage III–IV TTTS [11]. However, research also indicated that the risk of developing RVOTO does not differ in any Quintero stage [12]. Recipient twins in cases of stage IV TTTS are more likely to develop cardiomyopathy with right ventricle (RV) dilation and hypertrophy. The tricuspid valve regurgitation and aortic blood flow reversal through the ductus arteriosus in TTTS can cause the pulmonary valve flow to decrease, which can lead to narrowing and stenosis of the fetal pulmonary valve in the recipient twin. This may explain why stage IV TTTS is a risk factor for RVOTO. However, because cardiomyopathy occurs less often in TTTS donor twins, RVOTO has been reported less often in donor twins.

TTTS in which the recipient twin has RVOTO after laser therapy was documented as being associated with poorer outcomes [11]. In addition, high Quintero stage TTTS (stage III or IV) has been linked to poorer outcomes after FLP [30, 31], and 66.7%– 92.5% of TTTS cases in which the recipient twin has RVOTO have a high Quintero stage [11, 13, 29]. In our study, the percentages of high Quintero stage TTTS in the groups with and without recipient twins with RVOTO were similar (57.1% vs. 49.7%, respectively, *p* = 0.78); this similarity may explain why the TTTS group with recipient twins with RVOTO had comparable outcomes, including survival and normal cranial image rates, to those of the TTTS group with recipient twins without RVOTO after FLP. Through this study, we discovered that TTTS in which the recipient twin has RVOTO was not associated with higher incidence of neonatal ultrasound brain image anomalies. In our previous study, we demonstrated that the key predisposing factor for severe brain imaging anomalies in survivors of TTTS after FLP was younger gestational age at delivery [32]. Another report stressed that survivors with older gestational age at the time of FLP, younger gestational age at delivery, and lower birth weight have higher risk of developing neurodevelopmental impairment [33]. According to our data, the gestational age at delivery and birth weights of the donor and recipient twins did not significantly differ between the TTTS groups in which the recipient twin had versus did not have RVOTO (Table 1). This may explain the similarity in the brain image anomaly rates of the two TTTS groups.

RVOTO mainly comprises PA and PS; in our series, the incidence of PA in recipient twins with RVOTO was 14.3% (2/14). However, other studies have reported the risk of PA to be 28.5% (2/7) [10], 45% (24/53) [17], 19.2% (5/26) [9], and 42.8% (12/28) [11]. Therefore, the incidence of PA in recipient twins with RVOTO was relatively low in our study. PA has a poorer prognosis than PS does [34]; this may have contributed to the comparable outcomes of the TTTS groups with recipient twins with versus without RVOTO after FLP in our study.

The strength of this study is that it was based on data from a single center with consecutive cases. Operations were performed following the same surgical indications, and similar procedures and instruments were employed in all of the cases. The limitations of this study include the small number of cases, the low percentage of PA cases, and the possibility that the neonatal brain ultrasound results may not have revealed all cerebral injuries in the included neonates [32].

## Conclusions

In this study, the incidence of RVOTO in the recipient twins of cases of severe TTTS was 7.49%; Quintero stage IV and young gestational age at FLP were two risk factors for the recipient twin developing RVOTO in severe cases of TTTS. The perinatal outcomes (gestational age at delivery, perinatal survival rate, birth weight, and neonatal brain image anomaly rate) of the TTTS groups in which the recipient twin had versus did not have RVOTO after FLP did not significantly differ. This may have been because of the similar percentage of high Quintero stage in the two groups and the relative low percentage of PA in TTTS cases in which the recipient twin had RVOTO.

## Data Availability

The datasets obtained and analyzed in this study are available from the corresponding author upon reasonable request.

## Abbreviations

RVOTO: Right ventricle outlet obstruction
TTTS: Twin–twin transfusion syndrome
FLP: Fetoscopic laser photocoagulation
PS: Pulmonary stenosis
PA: Pulmonary atresia
TBV: Transcatheter balloon valvuloplasty
MVP: Maximum vertical pocket
IVH: Intraventricular hemorrhage
PVL: Periventricular leukomalacia
TOF: Tetralogy of fallot
